# Recent Advances in the Storage, Transportation, and Quality Control of *Eriocheir sinensis*: A Review

**DOI:** 10.3390/foods15142509

**Published:** 2026-07-15

**Authors:** Danni Zhao, Ruizhi Yang, Hanbin Lin, Lu Liu, Feng Yu, Peiying Shi, Bin Zheng, Rosana Moriana, Yadong Zhao, Soottawat Benjakul

**Affiliations:** 1 School of Food and Pharmacy, Zhejiang Ocean University, Zhoushan 316022, China; 2 School of Marine Biology and Fisheries, Hainan University, Haikou 570228, China; fengyu2022@hainanu.edu.cn; 3 Zhejiang Ocean Family Co., Ltd., Youpinyuan Road No. 1, Zhoushan 316000, China; 4 Research Group in Materials Technology and Sustainability (MATS), Department of Chemical Engineering, School of Engineering, University of Valencia, Avda. Universitat s/n, 46100 Burjassot, Spain; rosana.moriana@uv.es; 5 International Center of Excellence in Seafood Science and Innovation (ICE-SSI), Faculty of Agro-Industry, Prince of Songkla University, Songkhla 90110, Thailand

**Keywords:** *Eriocheir sinensis*, quality regulation, environmental stresses, waterless live transport, emerging technologies

## Abstract

The Chinese mitten crab (*Eriocheir sinensis*), as an aquatic food with high economic value, is favored by consumers for its high nutritional value and unique flavor. Its physiological status during temporary rearing and live transportation determines changes in meat quality, nutrition, flavor and food safety. Environmental stresses during this process are prone to inducing physiological disturbances in the crabs, resulting in reduced survival rates and degraded product quality. This paper systematically reviews the impacts of four pivotal environmental factors, namely dissolved oxygen (DO) levels, ambient temperature, water salinity, and ammonia nitrogen concentration, on the survival performance and quality maintenance of *E. sinensis* during temporary holding and live transport. It comprehensively clarifies the mechanisms through which fluctuations in these factors elicit physiological stress, oxidative damage, and metabolic disorders in the crabs while evaluating effective mitigation strategies such as gradient cooling and precise water quality regulation. In addition, key research gaps in the field are identified, including the lack of real-time vitality monitoring technologies and species-specific standardized transportation protocols, and future directions involving the integration of sensor technologies and adaptive environmental regulation are proposed. In summary, the survival status and quality of *E. sinensis* during temporary rearing and live transportation are synergistically regulated by various environmental factors. Developing scientific strategies can effectively mitigate the adverse effects of environmental stress, ensure food safety, and provide a theoretical basis for the live preservation, processing, and transportation of aquatic products.

## 1. Introduction

The Chinese mitten crab (*Eriocheir sinensis*) is a crustacean used for freshwater aquaculture [1]. Its aquaculture scale is unique, differing from that of widely distributed species such as the river shrimp [2]. It plays a core role in maintaining the balance of the water ecosystem [3] and also exhibits environmental adaptation characteristics in aquaculture. These characteristics reflect the application value and development potential in sustainable aquaculture [4]. From the perspective of food quality, given that *E. sinensis* is rich in nutrients and has a distinctive flavor, the live storage and transportation phase between harvest and processing plays a decisive role in food nutrition and safety [5]. At the same time, its edible pulp is rich in high-quality protein, essential fatty acids and various minerals, resulting in a demand that is relatively stable, especially in Southeast Asia [6,7,8,9].

**Figure 1:**
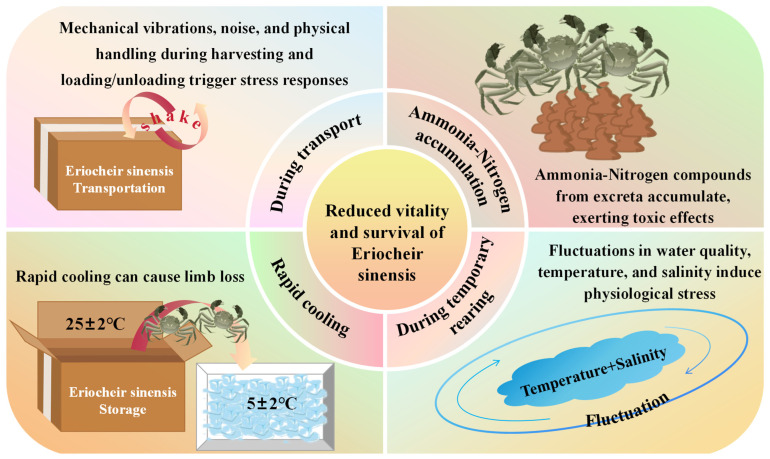
Stress response mechanisms of *E. sinensis* and their effects on survival performance and product quality during harvesting, temporary rearing and transportation [10,11,13,21,31].

As shown in Figure 1, *E. sinensis* endures persistent stress in the post-harvest supply chain, with its physiological stress regulated by temperature, cooling rate, dissolved oxygen (DO), water quality and salinity [10,11]. As a typical poikilothermic crustacean, it needs a suitable thermal environment to maintain metabolic balance, optimize energy distribution and inhibit the oxidative stress pathway. Fluctuations in temperature will break the balance: high temperature will interfere with the transmission of neural signals in young crabs [12]; sudden cooling will lead to osmotic and metabolic disorders; gradual cooling is helpful for physiological adaptation and reduces stress damage [13,14]. Hypoxia stress damages the mitochondrial respiratory chain of *E. sinensis*, hindering its growth, perhaps because reactive oxygen species (ROS) lead to death [15,16]. Poor water quality causes chronic stress, and the metabolites, abnormal carbon dioxide (CO_2_), ammonia nitrogen and pH value of *E. sinensis* form compound stress [17,18,19]. Salinity regulates the physiological state of *E. sinensis* through aspects such as osmosis, energy, and flavor. Raising *E. sinensis* at 7‰ salinity for 15 days can increase the umami substances in the muscle and optimize the flavor [20]. When studying the core stress pathways, it has been observed that different mechanisms are used by stressors at each stage to cause physiological damage. This metabolic process consumes a large amount of energy, often leading to reduced vitality and lower survival thresholds [21].

By modulating molecular and physiological indicators at a controlled rate, gradient cooling technology works in synergy with precise salinity control to alleviate stress. This approach not only preserves the quality and flavor of the crabs but also enhances their survival rate [22]. It is implemented in three stages, and its survival rate is much higher than that of traditional methods; it can also be finely adjusted according to the transportation time and the specifications of the crabs, with ecological and economic benefits [23]. At the same time, the environmental regulation layer uses technologies such as industrial cooling and keeping alive [24], efficient ammonia nitrogen removal [25], and precise CO_2_ concentration regulation [26]; nutritional regulation adds functional substances such as yeast culture and astaxanthin [27], activates the antioxidant system, reduces stress damage, and improves the post-harvest survival rate. There are common challenges in industrializing this technology, such as insufficient temperature control precision and high cost. The anhydrous live transportation technology lacks a standard system, and vitality monitoring is not sufficient. In the future, efforts need to be made in equipment innovation, process optimization, and customized model development to solve problems and promote standardization and large-scale application so as to contribute to the development of live aquatic product transportation and the *E. sinensis* logistics industry [28,29].

The sustainable and high-quality development of the *E. sinensis* industry relies heavily on a post-harvest storage and transportation supply chain system characterized by low losses and high stability. Building upon the invaluable foundations laid by previous research, this review aims to further extend and enrich the current understanding of crustacean preservation. Specifically, on the basis of the comprehensive review of single physical environmental stressors established by Fotedar, this work further expands the scholarly scope into a multifactorial interactive stress network, such as the hyperthermia–hypoxia metabolic conflict and salinity–pH ionic uncoupling, that is specifically calibrated for *E. sinensis* [21]. Furthermore, complementing the critical insights by Lin regarding the phenomenological patterns of muscle quality deterioration during processing and storage, this review enriches the existing literature by introducing a holistic linkage that maps upstream molecular–metabolic homeostasis directly onto downstream logistics engineering controls [30]. The core originality of this review lies in its pioneering construction of a ‘Trinity’ synergistic regulatory system, which integrates environmental adaptation, nutritional intervention, and physiological homeostasis into a unified closed-loop chain for waterless transport. At the same time, the lack of an industrialized precision–maintained-alive framework integrated with intelligent non-destructive testing technology makes it difficult to meet the industry’s needs for precise maintenance of live *E. sinensis* and targeted quality control. Based on these research gaps, this review uses *E. sinensis* as a representative model and focuses on the characteristics of composite stress caused by waterless storage and transportation with gradual cooling after harvest. Moving beyond the limitations of single-factor analysis, it innovatively establishes strategies for environmental adaptation and regulation, targeted nutritional intervention, and maintenance of physiological homeostasis, thereby constructing a three-pronged synergistic regulatory system. Unlike previous studies that merely described oxidative stress-induced physiological damage or the resulting deterioration in muscle quality, this review further integrates the concept of digital monitoring via intelligent sensor-based non-destructive testing to deeply analyze the regulatory mechanisms of composite environmental stress on the physiological activity, nutrition, and quality safety of crabs. It summarizes the application potential of new green storage and transportation technologies and outlines the future direction of integrated development between smart cold chains and the storage and transportation of fresh and live aquatic products. This review refines the framework for multifactorial environmental stress, establishes a precision environmental regulation approach based on physiological stress, and addresses research gaps in existing reviews regarding the mechanisms of combined stress and digital storage and transportation solutions. It provides scientific guidance and theoretical support for the green and efficient storage and transportation of crustacean aquatic products, as well as for reducing losses and enhancing efficiency in the industry.

## 2. Literature Search Strategy

To systematically review research progress on the anhydrous storage and transportation of *E. sinensis*, this review conducted targeted literature searches and screenings using the Web of Science, ScienceDirect, and PubMed databases. The search strategy employed a Boolean combination of three categories of search terms: terminology related to the subject organism (e.g., “crustaceans,” “*E. sinensis*”); terms related to environmental stress and transport conditions (e.g., “environmental stress,” “low-temperature stress,” “gradient cooling,” “waterless transport,” “waterless live-keeping,” “live transport,” “transport mode”); and terms related to physiology, metabolism, and quality regulation (e.g., “physiology and metabolism,” “metabolic regulation,” “oxidative stress,” “antioxidant capacity,” “quality changes,” “storage and transport quality,” “quality management”).

This review followed a standardized literature screening process, focusing primarily on peer-reviewed research articles and authoritative reviews published between 2010 and 2026, with an emphasis on selecting cutting-edge findings from the past five years. Inclusion criteria limited the study subjects to live *E. sinensis* or crustaceans; experimental treatments included storage and transport stress conditions such as gradual cooling, low-temperature stress, and anhydrous viability maintenance; research content centered on physiological metabolism, oxidative stress responses, and quality regulation; and the experimental systems were comprehensive, with detailed data. We excluded studies with inappropriate subjects, those lacking relevant stress or storage and transportation treatments, and irrelevant literature focusing on aquaculture nutrition or product processing. We also excluded low-quality literature such as reviews, popular science articles, patents, duplicate publications, and studies with incomplete data. A comprehensive and standardized literature search process ensured the comprehensiveness, timeliness, and representativeness of the included studies, providing ample and reliable literature support for this review to systematically organize different storage and transportation technologies and quality control measures, conduct critical comparisons of their advantages and disadvantages, and identify gaps in existing research as well as future directions.

## 3. Factors Influencing Physiological Stress in *Eriocheir sinensis*

Physiological stress in *E. sinensis* specifically denotes a series of systemic physiological imbalances triggered when environmental factors deviate from optimal ecological thresholds during temporary holding and live transport. These imbalances manifest primarily as metabolic disorders, oxidative damage, and immune suppression, ultimately leading to significantly reduced post-harvest survival rates and severe deterioration in commercial quality [30,32,33]. This section focuses on water quality parameters such as temperature, dissolved oxygen, ammonia nitrogen, CO_2_, pH, and salinity and examines the environmental stress effects caused by various water quality factors. It systematically analyses the molecular mechanisms by which these factors induce stress responses through specific physiological regulatory pathways, clarifying the synergistic effects between different environmental factors. This provides a robust theoretical foundation for the subsequent development and optimization of a stress regulation technology system for post-harvest *E. sinensis*.

### 3.1. Temperature: A Core Driver of Metabolic Regulation and Stress Responses

**Figure 2:**
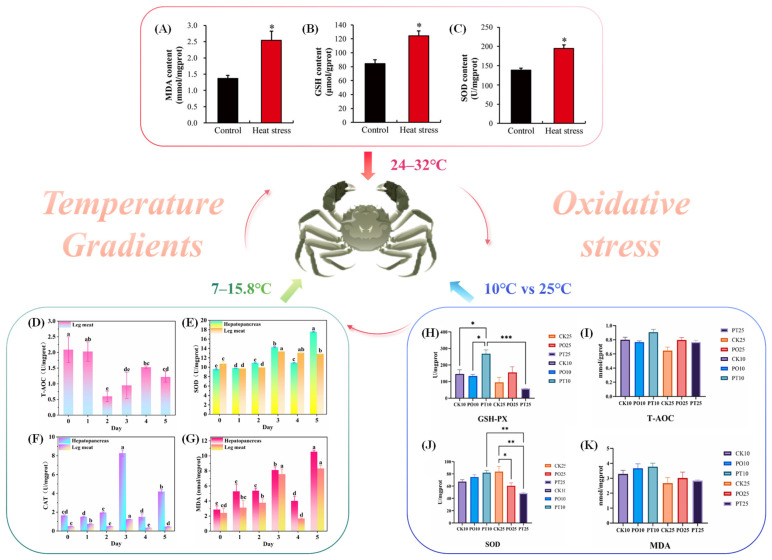
Trends in metabolic rate and oxidative stress markers under different temperatures [12,39,40]. (**A**–**C**) represent, respectively: Effect of heat stress on antioxidant capacity of juvenile *E. sinensis*. (**A**): MDA; (**B**): GSH; (**C**): SOD. “*” shown above the columns represent signifi cant differences (* *p* < 0.05). (**D**–**G**) represent, respectively: Changes in T-AOC (**D**) in leg meat and SOD (**E**), CAT (**F**) and MDA (**G**) levels in hepatopancreas and leg meat on different days. Different letters in each parameter indicate significant differences across days (*p* < 0.05). (**H**–**K**) represent, respectively: Differences in antioxidant parameters among groups of *E. sinensis* treated at different temperatures and exposure concentrations ((**H**): GSH-PX; (**I**): T-AOC; (**J**): SOD; (**K**): MDA). Note: The temperature gradients are 10 °C and 25 °C; the concentrations of polystyrene nanoparticles were 0, 1, and 10 mg/L, respectively. CK10 (0 mg/L, 10 °C), CK25 (0 mg/L, 25 °C), PO10 (1 mg/L, 10 °C), PO25 (1 mg/L, 25 °C), PT10 (10 mg/L, 10 °C) and PT25 (10 mg/L, 25 °C). In each figure, the asterisk (*) label indicates a statistically significant difference between different treatment groups. * Indicates significant *p* < 0.05, ** indicates significant *p* < 0.01, *** indicates significant *p* < 0.001. Note: Reprinted with permission from Zhang, C., Journal of Thermal Biology; Lin, H., Food Chemistry: X; and Zhou, Z., Journal of Hazardous Materials.

*E. sinensis* is a poikilotherm. Its metabolic rate and enzyme activity are precisely regulated by temperature. This makes it a major factor causing physiological stress, such as energy balance disruption [34]. When the temperature rises from 24 °C to 32 °C at a rate of 1 °C per hour, it accelerates the basal metabolic rate, leading to an energy imbalance and excessive energy expenditure [12]. At this point, the nuclear factor erythroid-derived 2-like 2 (Nrf2)/glutathione peroxidase (GPx) signaling pathway is significantly upregulated, becoming the primary defense mechanism against oxidative stress, while superoxide dismutase (SOD) activity is inhibited and impaired [35]. This directly triggers the oxidative stress cascade reaction, causing a significant increase in the oxidative stress level of the organism. Especially under drought stress, the higher temperature will further aggravate the problem of physiological function, making the mortality rate show a rapid upward trend [36]. As shown in Figure 2, the metabolic rate (measured by oxygen consumption) of crustaceans and oxidative stress biomarkers (such as malondialdehyde (MDA) and SOD) exhibit distinct response patterns across temperature gradients.

Sudden exposure to a cold environment of 15 °C can trigger an acute stress response and then disrupt the ion balance and physiological homeostasis [14]. Conversely, gradual cooling can provide an adaptive window for organisms and reduce the stress damage by regulating membrane lipid fluidity and enzyme activity. For example, the rapid cooling during live transportation can lead to mechanical injuries such as the shedding of crustacean limbs. Using pre-cooling sawdust/wood shavings as buffer packaging can reduce cold stress and physiological damage [13]. The appropriate low-temperature environment can induce *E. sinensis* dormancy. Putting *E. sinensis* into a dormant state can reduce the accumulation of hepatopancreas metabolites and the overall metabolic situation, alleviate the impact of air exposure stress, and improve the survival rate after harvest. It is found that the threshold of cold anesthesia (CA) dormancy of *E. sinensis* is −2 to 10 °C. Under the CA condition of 7 °C, the maximum survival time can reach 134 h [37].

Empirical data show the key regulation of temperature. When the ambient temperature rises from 7 °C to 17 °C, the oxygen demand of *E. sinensis* increases by 10.5 times [38], increasing the physiological burden and metabolic pressure. Therefore, temperature is the core environmental factor controlling the metabolic activity and physiological pressure of *E. sinensis*. In different environmental scenarios, there are obvious intraspecific variations in the physiological effects of temperature on the larvae and adults of *E. sinensis*. However, existing temperature-related studies still have significant limitations. Most studies have only investigated temperature stress in *E. sinensis* of a single size and growth stage, lacking research on the optimal temperature ranges for different sizes, growth stages, and physiological states. A comprehensive understanding of how temperature affects the entire growth cycle has not yet been established, and existing studies on temperature regulation lack validation relevant to practical applications. Therefore, research on growth characteristics, body size, and precise temperature control can effectively alleviate the physiological stress experienced by crabs during post-harvest storage and transportation, providing a key breakthrough for improving the post-harvest survival rate and quality of *E. sinensis*.

### 3.2. DO: A Key Regulatory Factor in Redox Equilibrium

DO, serving as the core substrate for respiratory metabolism in *E. sinensis*, directly mediates cellular redox homeostasis regulation and energy supply pathways (such as ATP synthesis). When concentrations range from 6.5 to 12.5 mg/L, the metabolic rate increases significantly, making concentration fluctuations a key environmental signal that induces physiological stress in this organism [41]. Hypoxic stress first disrupts redox homeostasis, subsequently triggering cascading damage effects: inducing neurotoxicity [42], compromising intestinal mucosal barrier integrity [15], and initiating mitochondrial-mediated apoptosis [43]. Ultimately, this comprehensively impairs nutrient absorption efficiency and immune defense functions.

The effects of hypoxia on the body are concentration and time-dependent. Short-term moderate hypoxia activates compensatory mechanisms, which can be demonstrated by the increase in total antioxidant capacity (T-AOC) and the enhancement of SOD and catalase (CAT) enzyme activities [44]. Hypoxia is a stress factor. When the DO in the aquaculture water suddenly drops, the behavioral rhythms of aquatic crustaceans and other situations will be disordered [15]. Long-term hypoxia will also damage the hepatopancreas and gills and inhibit growth and development [45].

Crustaceans are extremely sensitive to low-oxygen conditions. The sudden appearance or long-term existence of low DO in the culture pond can cause tissue damage or death [16]. Transcriptomic studies of crab gills under hypoxic conditions have confirmed that hypoxic stress alters the expression of genes associated with respiratory metabolism and stress, indicating that dissolved oxygen is a key environmental factor determining the physiological homeostasis and survival of crustaceans. Fluctuations in DO concentration can cause organisms to transition from short-term adaptation to a state of sustained damage by regulating their redox and energy metabolism. However, existing studies still have significant limitations. Most hypoxia studies employ static treatments under constant low-oxygen conditions, making it difficult to simulate dynamic stresses such as declining DO gradients and intermittent hypoxia encountered during live storage and transport. Furthermore, these studies primarily focus on short-term acute hypoxia and lack research on hypoxia thresholds for crabs of different sizes and growth stages; they also fail to incorporate the regulation of dissolved oxygen levels during actual storage and transport dynamics. Future research should further refine a multidimensional hypoxic stress system to provide a theoretical foundation for post-harvest stress regulation techniques in *E. sinensis*.

### 3.3. Synergistic Stress Effects of Ammonia Nitrogen, CO_2_ and pH

**Figure 3:**
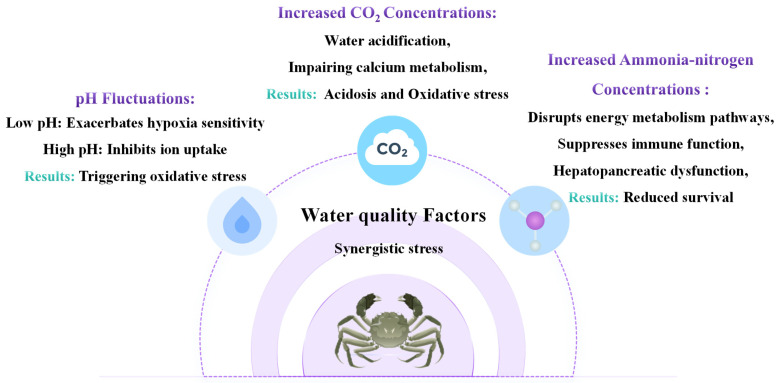
Ammonia nitrogen, CO_2_, and pH levels collectively exacerbate physiological stress [18,48,49,52].

Ammonia nitrogen, CO_2_, and pH are key water quality factors regulating the post-harvest physiological homeostasis of *E. sinensis*. Figure 3 shows that the dynamic imbalance of these three parameters will destroy the stability of the internal microenvironment and jointly trigger physiological stress, and their synergistic stress will also aggravate physiological damage. From the perspective of pollution sources, ammonia nitrogen is the main endogenous pollutant in the temporary culture water body, coming from the excretion of animal metabolic nitrogen, the decomposition of biological residues, and the corruption of fecal residues. Short-term low-concentration ammonia nitrogen is a metabolic by-product and does not cause obvious ammonia stress to *E. sinensis* [25]. However, when the water pH reached 7.69 ± 0.46, and the ammonia nitrogen concentration reached 41.87 mg/L, these levels exceeded the tolerance range of the organisms. At this point, α-D-glucose levels rose significantly, immune function was markedly suppressed, and energy metabolism homeostasis was disrupted, leading to hepatopancreatic damage and a significant reduction in the post-harvest survival rate of the cultured crabs [18].

High concentrations of CO_2_ can lead to ocean acidification. This disrupts calcium metabolism in crustaceans, inducing acidosis and oxidative stress and jeopardizing the normal processes of molting and exoskeletal mineralization [17]. When the pH of seawater drops to 7.0, the survival rate of *Scylla serrata* decreases significantly by 30%; at a pH of 7.3, *Horseshoe Crab* exhibits symptoms of oxidative stress and acidosis. At the same time, its behavioral activity is suppressed, and alkaline phosphatase activity decreases during bone mineralization [46,47]. This stress situation also occurs in *E. sinensis*.

The fluctuation of pH value makes the stress damage caused by ammonia nitrogen and CO_2_ more serious. A low pH value will make the hypoxia stress more prominent [48], and a high pH value will hinder the transport and absorption of ions by gill tissues [49]. These two mechanisms break the ion balance and thus lead to oxidative stress. Crustaceans have specific pH tolerance ranges. *E. sinensis* is acidic when the pH value is 6.5 ± 0.20 [19]; prolonged exposure of juvenile shrimp to extreme pH conditions ranging from 6.5 to 9.5 also markedly impedes their growth and developmental processes [50].

The synergy of various water quality factors leads to the occurrence of “1 + 1 > 2” synergistic damage. Studies on crustacean metabolism have confirmed that the combined stress of high ammonia nitrogen and high CO_2_ can cause more severe damage to the hepatopancreas than single stress [25,47]. Fluctuating ammonia nitrogen, CO_2_, and pH values disrupt the balance in crustaceans and jointly trigger physiological stress [51]. Multiple water quality factors act synergistically to exacerbate tissue damage, directly reducing the post-harvest survival rate of *E. sinensis* during storage and transport, thereby providing a basis for developing water quality control strategies. However, research on combined water quality stress has not accounted for the dynamic environmental impacts of gradual accumulation and gradient changes in water quality during storage and transport. Furthermore, an industrial-scale model for the synergistic regulation of multiple water quality factors during storage and transport has yet to be established, and post-harvest stress-relief and survival-enhancement technologies still require further refinement.

### 3.4. Salinity: A Balancer Factor in Osmoregulation and Energy Allocation

Salinity is a key environmental factor that regulates the post-harvest physiology of *E. sinensis*. It controls the osmoregulatory pathway and energy distribution pattern and dynamically regulates its stress response. When the salinity abnormally lowers the osmotic environment, *E. sinensis* quickly activates the compensatory mechanism: it enhances glycogenolysis to increase the basal metabolic rate, activates the fat decomposition pathway to provide energy, and also relies on hemoglobin degradation to maintain osmotic balance. This process redirects the energy originally used for growth to the maintenance of osmotic homeostasis, forming the “energy competition between growth and stress response” [22]. At the same time, the nutritional needs of *E. sinensis*, such as protein, carbohydrates, lipids, and minerals, will adapt to the fluctuation of salinity [53].

Crustaceans have species-specific salinity tolerance thresholds. A study on *Macrobrachium rosenbergii* shows that when the salinity is ≤10 ppt, the physiology is in a normal state, and when the salinity is 20 ppt, the mortality rate reaches 50% within 72 h [54].

For *E. sinensis*, holding within the optimal salinity range yields the dual effects of “flavor regulation + nutritional enhancement”: on the one hand, it intensifies characteristic flavor intensity by modulating non-volatile flavor compounds [55], not only ensuring osmotic homeostasis but also significantly increasing docosahexaenoic acid (DHA), eicosapentaenoic acid (EPA), total essential free amino acids (∑EFAA), total free amino acids (∑FAA), and total umami value (∑TUV), thereby improving palatability [56].

Abnormal salinity leads to quality degradation and causes stress damage. During live fish transportation, unsuitable salinity accelerates lipid oxidation and protein degradation, resulting in an unpleasant fishy smell. Take dimethyl sulfide flavored by chars as an example; there is a concentration-dependent relationship between its concentration and flavor perception: low concentration endows *E. sinensis* with a unique fresh taste, and high concentration releases a pungent sulfur flavor [57]. From the molecular mechanism, both excessively high and low salinity activate the expression of osmoregulatory genes, increasing physiological metabolic costs. The dynamic energy budget–toxicokinetic–toxicodynamic (DEB-TKTK) model, grounded in dynamic energy budget theory, predicts that 60 days of holding in an 18‰ salinity environment can synchronously enhance both osmotic regulation capacity and antioxidant stress resistance in *E. sinensis* [58].

Specifically, the appropriate salinity enables the accumulation of flavor substances such as umami amino acids and enhances the antioxidant capacity [58] and also improves the flavor of the edible part. This indicates that salinity has a dual regulatory role in alleviating stress and improving quality. Salinity regulates the stress response of *E. sinensis* through osmotic regulation and energy allocation. Optimal salinity influences physiological costs, post-harvest survival rates, the accumulation of flavor compounds, nutritional quality, and gonadal development. Deviations from the optimal range increase osmotic metabolic stress and exacerbate physiological stress-related losses. Most existing studies focus on energy allocation under constant, steady-state salinity conditions, neglecting the monitoring of osmotic dynamics under dynamic stresses—such as sudden salinity drops and salinity gradients—that occur during actual post-harvest storage and transportation. Consequently, these studies are unable to quantify instantaneous energy expenditure and metabolism. Furthermore, many studies are limited to single-factor treatments, making it difficult to explain the synergistic amplification of stress damage under multifactorial stress conditions.

### 3.5. Multifactorial Interactions: Complexity of Cumulative Stress

In practical aquaculture and post-harvest handling scenarios involving *E. sinensis*, environmental stressors rarely act upon the organism in isolation. The compound stress effects arising from their interactions frequently exacerbate stress cascade responses and amplify physiological damage, highlighting the systemic and complex nature of synergistic multifactorial environmental influences. The synergistic stress effects between typical environmental factors are mainly reflected through three core modes, and each mode causes damage by disrupting the homeostasis of energy metabolism:

Scenario 1: Synergistic metabolic conflict between hyperthermia and hypoxia: Elevated temperatures markedly accelerate the basal metabolic rate in crustaceans, precipitating a steep increase in mitochondrial oxygen consumption. Hypoxic stress blocks the activity of respiratory chain complexes, and the ATP production pathway stops. The combination of temperature rise and hypoxia not only destroys the activity of core antioxidant enzymes but may also lead to an increase in blood sugar. This promotes the production of lactic acid (LA), the rapid accumulation of LA anaerobically, and finally, the pH value of hemolymph changes. This change in turn regulates the core physiological mechanisms and metabolic processes of crustaceans [59,60]. This leads to the metabolic contradiction of a “sharp increase in oxygen demand and disruption of energy supply” and finally results in severe energy metabolism disorder and oxidative stress damage [15,34].

Scenario 2: Synergistic effects of salinity and pH on ionic equilibrium: Osmotic stress induced by abnormal salinity produces a “1 + 1 > 2” synergistic injury when pH deviates from the optimal range. pH fluctuations damage the structure of ion channels in the gill epithelium and inhibit the activity of Na^+^/K^+^-ATPase. The combined action of salinity and pH fluctuations not only disrupts ion balance but also induces oxidative stress damage and may also lead to metabolic disorders and increased energy consumption. Environmental stress factors in aquatic ecosystems make metabolic activities stronger and maintain homeostasis through compensatory mechanisms. This makes the ion imbalance caused by salinity more serious, which greatly increases the cost of osmoregulation and aggravates physiological stress [19].

Scenario 3: Synergistic energy depletion effects of CA and water deprivation (WD) duration: CA inhibits metabolic enzyme activity, while water deprivation blocks gill respiration. The combined stress initiates the anaerobic glycolysis pathway. A large amount of LA is produced in muscles, and acidosis occurs in the body. The abnormally high activity of ATPase aggravates energy consumption and finally damages the hepatopancreas tissue [36,61].

The aforementioned studies confirm that the interaction of environmental stressors is directly linked to energy metabolism disruption and post-harvest survival rates (Table 1). This characteristic indicates that environmental management for *E. sinensis* must abandon single-factor regulation approaches, instead establishing a comprehensive regulatory system based on “multi-factor synergistic adaptation” (Table 2). When formulating environmental management measures for breeding ponds and transportation, it is necessary to systematically consider the relationships between various factors so as to accurately predict and control stress damage.

## 4. Gradient Cooling Technique for Waterless Live Transport of *Eriocheir sinensis*

As a high-value freshwater crustacean possessing both nutritional and economic significance, *E. sinensis* is primarily transported post-harvest via either frozen or live transport methods. While frozen transport offers advantages such as extended shelf life and lower costs, quality deterioration frequently occurs during freezing and storage, manifesting as juice loss, protein oxidation, and lipid oxidation [24,63,64]. In contrast, live transport maximizes the preservation of nutritional value and flavor characteristics, aligning with consumers’ growing demand for fresh aquatic products. Consequently, developing efficient waterless live transport technology for *E. sinensis* is crucial for maintaining their post-harvest quality and market value.

### 4.1. The Necessity of Gradient Cooling Technology: Addressing Core Stress Challenges in Live Transport

The physiological stress experienced by *E. sinensis* during live transport without water is primarily induced by the synergistic effects of three major factors: environmental fluctuations, mechanical damage, and improper handling. The large change in temperature is a key stressor [65]. The moist shavings in the container can maintain humidity and reduce friction to alleviate stress but cannot solve the problem of temperature fluctuation [66].

Existing conventional temperature-controlled live-storage technologies, such as pre-cooling and CA storage, have significant limitations: they can only specifically regulate a single stress factor to alleviate localized stress but cannot block temperature-induced stress pathways at their source (Table 3). Numerous studies on crustaceans have confirmed that the progressive temperature rise treatment of *Macrobrachium rosenbergii* will induce environmental temperature stress and then interfere with hormone regulation. Heat stress will also affect the level of norepinephrine in the hemolymph, thus affecting immunity [67]. Following CA treatment in cold sawdust, the overall appearance, color, and flavor of the flesh of *Penaeus monodon* showed significant improvement compared to freshly caught dead prawns. The optimal cooling gradients required for different storage and transportation cycles vary, making it difficult to apply a uniform set of parameters [68]. Currently, commonly used auxiliary life-support measures—such as low-temperature anesthesia and oxygenation interventions—can only alleviate local or temporary stress; they lack a comprehensive, systematic, steady-state regulation system, making it difficult to achieve systematic prevention and control throughout the entire process. In contrast, gradient cooling technology establishes a continuous temperature adaptation window, addressing this shortcoming; it promotes the entry of *E. sinensis* into a state of CA and dormancy, thereby mechanically interrupting the stress cascade triggered by sudden temperature changes. This achieves the dual effects of extending survival duration and improving survival rates, providing an effective solution to the core challenge of waterless storage and transportation.

### 4.2. Mechanisms of Gradient Cooling: A Controlled Adaptation Pathway for Stress Alleviation

The core physiological basis of gradient cooling is the adaptive mechanism for temperature gradients, which is closely related to the poikilothermic nature of *E. sinensis*. *E. sinensis* lacks the ability to regulate its body temperature autonomously; its core body temperature remains in dynamic equilibrium with the ambient temperature at all times. The rate of change in ambient temperature directly determines the intensity of its physiological stress, and the cooling rate is significantly positively correlated with the organism’s stress level and the degree of physiological disruption [75].

Gradual cooling allows the ambient temperature to approach the low-temperature dormancy threshold at a controlled rate, prompting the body to initiate a stepwise adaptive regulatory process. By regulating cell membrane lipid fluidity and the activity of key metabolic enzymes, the body achieves phased physiological adaptation, gradually entering a state of low-metabolic dormancy. This, in turn, lowers heart rate and basal metabolic rate, thereby reducing the body’s energy expenditure at its source [76]. A gradient cooling rate of 1–3 °C/h maintains metabolic regulation stability and prevents metabolic disorders and excessive energy consumption [37]. Compared with the sharp fluctuations in oxygen consumption caused by rapid cooling, gradual cooling steadily reduces the organism’s metabolic demands while inhibiting the activity of glycogen phosphorylase, thereby effectively protecting the organism’s glycogen reserves. Since glycogen is the primary energy source for crabs under low-temperature stress, the stability of its reserves directly determines the organism’s stress resistance and survival [34,61].

Gradual cooling can mitigate cellular damage caused by acute cold stress and reduce the peak intensity of the stress response by precisely regulating the timing and intensity of stress pathway activation. At the molecular level, acute temperature fluctuations significantly induce the overexpression of heat shock protein 70 (HSP70) and glutathione S-transferase at both the genetic and protein levels, triggering a sustained oxidative stress cascade and causing irreversible oxidative damage; in contrast, a gradual cooling regimen significantly attenuates the abnormal overexpression of these stress molecules and blocks the sustained transmission of oxidative damage signals [15,77]. At the physiological regulation level, the low-temperature microenvironment created by gradient cooling collaboratively alleviates the physiological disorders caused by salinity fluctuations: on the one hand, it stabilizes the osmotic regulation pathway, alleviates the imbalance of the antioxidant system, and inhibits cell apoptosis [78]; on the other hand, it maintains the steady-state activity of Na^+^/K^+^-ATPase in gill tissues, ensures ion transmembrane transport, and prevents cell damage caused by osmotic imbalance [24]. At the metabolic level, LA serves as an important lipid energy reserve and a key metabolic product in the hepatopancreas of *E. sinensis*; metabolic disorders involving LA can inhibit mitochondrial respiratory chain function and induce oxidative stress damage [36]. Gradual cooling can maintain metabolic homeostasis in the liver and pancreas by systematically lowering the overall metabolic rate, thereby effectively preventing secondary oxidative stress caused by energy metabolism imbalances.

In summary, graded cooling induces phasic cold-adaptive regulation in *E. sinensis*, leading to the formation of multiple physiological homeostatic mechanisms: by reducing the metabolic cost of compensation, it protects core energy reserves such as glycogen and lipids; by attenuating the abnormal activation of stress signaling pathways, it alleviates oxidative damage at the cellular and molecular levels; and by stabilizing osmotic and metabolic balance, it counteracts the synergistic stress effects caused by multifactorial coupling, thereby establishing a comprehensive regulatory system for the organism’s stress response.

### 4.3. Implementation Process of Gradient Cooling: A Three-Stage Standardized Protocol

**Figure 4:**
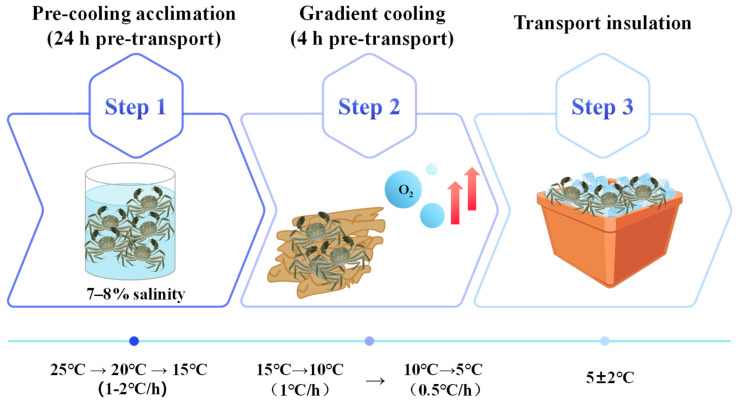
A three-stage coordinated gradient cooling method based on the integration of holding and transport phases. Note: The “→” symbol in the figure indicates that the temperature decreases sequentially.

Temperature control is a critical factor in the post-harvest supply chain for the storage and transportation of *E. sinensis* without water. Numerous studies have demonstrated that gradient cooling can effectively alleviate physiological stress in the crabs and improve survival rates during storage and transportation. Based on existing research on environmental temperature factors, a conceptual three-stage gradient cooling process has been developed (Figure 4). Currently, all parameters are derived from static simulation storage and transport experiments; no industrial protocols have been established yet, and the stability of the process requires further validation. The aim is to establish a continuous control chain for waterless storage and transport through segmented, progressive cooling, ensuring continuous and stable operation at each adjustment stage.

The first stage is called the “pre-cooling adaptation period”, which starts 24 h before the conversion. The main purpose is to facilitate the transportation of *E. sinensis* through low-temperature acclimation. Pre-transport cooling alleviates transport stress, reduces mortality, and can maintain the muscle yield and the vitality of the body during subsequent processing. Also, a relatively low cooling rate can improve the health score after transportation [69]. The standardized operation in this stage is as follows: in the temporary rearing pond, reduce the water temperature from the basic breeding temperature (20–25 °C) to the target pre-cooling temperature of 15 °C at a rate of 1–2 °C/h and keep the salinity at 7–8‰ to improve the anti-stress ability [20]. The core mechanism enables the expression level of cold-adaptive genes to be upregulated, thus creating physiological adaptability for deep cooling and anhydrous transportation.

The second stage is called “deep cooling”, starting 4 h before transportation. It involves gradual gradient cooling to reduce the core temperature of *E. sinensis* so as to inhibit metabolic activities during transportation. This stage includes two substeps: Step 1: Reduce the crab’s body temperature from 15 °C to 10 °C at a rate of 1 °C per hour within 2 h. At this time, the crabs are placed in moist wood shavings with a humidity of 85%. This procedure induces a semi-dormant state in the crabs, reducing their heart rate from 30 to 15 beats per minute, and the metabolic activity also decreases accordingly [36]. Step 2: Reduce the temperature by 0.5 °C per hour for 2 h, bringing the temperature of the crab from 10 to 5 °C. At this time, it is necessary to supplement low-concentration oxygen, with a concentration of 5 to 8 mg per liter, to avoid the activation of anaerobic glycolysis. Such an operation can reduce glycogen consumption by half, thereby saving the core energy reserve [61]. Precisely adjust the cooling rates in the two substeps to achieve a smooth transition from the semi-dormant state to the deep metabolic inhibition state and avoid the stress rebound caused by temperature changes.

The third stage is the stage of transportation and insulation. Its core is to maintain low-temperature stability starting from pre-cooling. The purpose is to reduce the impact of temperature fluctuations during transportation on *E. sinensis*. The ambient temperature is controlled at 5 ± 1 °C by using phase-change materials, and the fluctuation range is within ±2 °C. This technology can prevent rewarming stress [24]. Short-term fasting can balance the energy metabolism and ammonia nitrogen stress of crustaceans, inhibit cell apoptosis, and maintain/promote muscle mass [79]. The green and economical waterless fresh-keeping technology with pre-cooling as the core can optimize the transportation efficiency. This technique induces a dormant state in crabs through low temperatures, enabling them to maintain core physiological vitality in an anoxic environment. Its economic and environmental benefits are in line with the principles of sustainable development and also in line with the current trend of aquatic live product transportation research [80].

At the same time, when implementing this gradient cooling process in industrial-scale storage and transportation, it is still necessary to be aware of the limitations regarding the applicable ranges of various parameters. During actual storage and transportation, the cooling duration, cooling rate, environmental humidity, and dissolved oxygen control thresholds should be adjusted based on the size and specifications of *E. sinensis*. For large-scale, long-distance simulated storage and transportation, complex industrial environments—such as dynamic environmental fluctuations—must be further considered; the relevant parameters should be used only as conceptual references for control.

### 4.4. Application Outcomes of Gradient Cooling: Dual Enhancement of Survival Rate and Quality

It is important to optimize the technology for waterless live transport of *E. sinensis* based on existing live storage and transport methods (Table 4). Crustaceans exhibit highly consistent physiological and metabolic characteristics under temperature fluctuations, hypoxia, and water deprivation. Under environmental stress, anaerobic glycolysis intensifies and lactic acid accumulates in large quantities; the body’s antioxidant system is activated to scavenge reactive oxygen species; the activity of enzymes involved in osmotic regulation fluctuates; immune function is suppressed; and stress signaling pathways such as Nrf2 and mTOR are simultaneously activated. Prolonged stress leads to tissue damage and protein degradation, which is the common underlying mechanism behind quality deterioration and increased mortality rates during the storage and transport of various crustaceans [30,81]. When transporting live *E. sinensis*, a treatment regimen involving a gradual temperature gradient from 25 °C to 7 °C, rather than transferring them directly to a 7 °C environment, significantly increased the transport survival rate to 80.6% and extended the survival duration for 50% of the individuals. Gradual cooling effectively alleviates oxidative damage to the organism and inhibits the decline in hemolymph pH, yielding superior results in maintaining physiological vitality and muscle quality [13,69]. Anesthesia with eugenol–tricaine before transportation increased the survival rate but aggravated muscle fiber rupture and tissue damage and greatly increased the transportation cost, presenting technical limitations [62]. Research on crustacean transportation involves anesthetizing *Panulirus ornatus* with 50 ppm isobutanol at 28 °C for a 22 h soak to inhibit metabolic activity to reduce hemolymph ammonia concentration, thereby reducing physiological stress and improving transportation survival rate [82]. For *Portunus trituberculatus*, adopting the method of “vacuum extraction–oxygen filling (4.8 L)–24‰ salinity seawater supplement (0.7 L)” results in a 24 h survival rate of about 80% [83]. When *Macrobrachium rosenbergii* is simulated to be transported without water for 32 h, CO_2_ is removed by adding limewater (Ca(OH)_2_ solution) in a foam box, and Best Management Practices (BMP) treatment is carried out to improve the microenvironment and increase the survival rate [23]. *Litopenaeus vannamei* is preserved by means of gradient cooling; for example, high-pressure cold plasma technology combined with the extract of Sophora japonica leaves can extend the shelf life to 15 days at 4 °C [84]. This technology can also be used together with natural preservatives (plant extracts, chitin oligosaccharides, bioactive peptides, and essential oils) [85] or act synergistically with natural antioxidants (for example, increasing the contents of bioactive peptides, chitin derivatives, and carotenoids) [86]. These methods prolong the fresh food transportation time and devise various technical approaches for quality maintenance in cold chain logistics. In general, gradient cooling technology has all-around advantages in the 48 h waterless live transportation of *E. sinensis*. It can improve the survival rate during transportation, reduce muscle damage, maintain muscle quality, stabilize vital signs, and maintain the freshness and flavor of crab meat, highlighting its double value in improving the survival rate and maintaining product quality.

### 4.5. Challenges and Future Directions

The gradient cooling technology has advantages in the dry transportation and live transport of *E. sinensis.* However, at this stage, there are still multiple bottlenecks in industrial applications, and existing research in this area has significant methodological limitations that hinder the large-scale implementation of the technology. In terms of industrial practice, the technology faces three major practical challenges: insufficient temperature control precision, seasonal fluctuations in crab size, and relatively high storage and transportation costs. In waterless live transport, temperature fluctuations of varying magnitudes (15 ± 1 °C, 15 ± 2 °C, 15 ± 3 °C) disrupt the homeostasis of crab muscle quality and physiological metabolic rhythms, suppress immune function, and ultimately lead to a decline in product quality [71]. Small-scale transportation is easily affected by external temperature changes, so high-precision temperature control containers (tolerance ≤ ±0.5 °C) are needed to ensure the technical effect [90]. During transportation, the physiological metabolism and growth rate of crab muscles change dynamically due to internal and external factors. This leads to differences in muscle morphology and individual quality among crabs of different sizes. For instance, juvenile crabs weighing less than 100 g need a cooling rate of 0.5 °C per hour, which is slower than the cooling rate of 1 °C per hour for adult crabs weighing more than 200 g [91]. Therefore, customized solutions based on size are needed. At the methodological level, the studies did not simulate the combined dynamic stresses encountered in actual logistics—such as fluctuations in temperature and humidity, mechanical vibrations, and the accumulation of gases and ammonia nitrogen—resulting in process parameters that are disconnected from industrial storage and transportation scenarios and thus have limited practical applicability. Additionally, existing studies generally suffer from small sample sizes and insufficient experimental reproducibility. Nevertheless, there is a high degree of consistency in the core conclusion that gradual cooling can mitigate oxidative damage during the storage and transportation of *E. sinensis* and improve their survival rate, confirming that this technology remains universally applicable.

To meet the challenges, future research needs to clarify the optimized pathways. In terms of green and safe management, combining inductively coupled plasma mass spectrometry (ICP-MS) with a reliable traceability model can monitor the health risks of trace elements in crab meat and also improve meat quality and food safety [92]; when optimizing costs, given that phase-change materials account for 15% of the transportation cost, the industry is carrying out related work on low-cost substitutes, such as using plant fiber insulation pads to control transportation costs [23]. In the future, it is necessary to integrate multidisciplinary methods to solve key technical problems and also adjust localized parameters to realize the standardized and large-scale application of gradient cooling technology in the *E. sinensis* industry. This can provide necessary technical support for the high-quality development of live aquatic product transportation.

## 5. Practical Application of Waterless Live Transport for *Eriocheir sinensis*

As a pivotal extension of gradient cooling technology in aquatic transport, waterless live transport operates on the core principle of inducing hibernation in *E. sinensis* through low temperatures. This precisely addresses three fundamental challenges prevalent in traditional aquatic transport: high energy consumption, high loss rates, and susceptibility to quality deterioration. This section examines the practical application framework of waterless live transport, focusing on how the synergistic regulation mechanism between gradient cooling technology and the waterless environment achieves three objectives: extending survival duration, stabilizing product quality, and reducing overall transport costs. It also systematically analyses key challenges encountered during large-scale implementation, including adaptability, economic viability, and standardization.

### 5.1. Core Mechanisms: Dormancy Induction and Metabolic Regulation

Anhydrous live transportation relies on gradient cooling to alleviate stress. Its core function is related to the physiological adaptive regulation of *E. sinensis* in low-temperature and anhydrous environments. When encountering cold stress, crustaceans initiate a diapause stress response and enter a semi-diapause or complete diapause state. In this way, the metabolic rate and energy consumption are greatly reduced, and the survival ability of organisms is significantly improved [23]. Studies on *E. sinensis* show that when it is placed in oxygenated water at 25 °C and cooled to 7 °C at a rate of 3 °C/h, the temperature range of CA dormancy is −2 to 10 °C, and 7 °C is the most suitable temperature in this state. Under this temperature, the maximum survival time of crabs can reach 10 days [37]. The step-by-step cooling program (from 25 °C to 15 °C to 5 °C) reduces the metabolic rate to below 30% of the normal level, the heart rate drops from 30 beats per minute to 8 beats per minute, and the consumption of liver glycogen is reduced by 60%, achieving precise regulation of core physiological indicators [36,61]. In related studies on the *Penaeus monodon Fabricius*, researchers employed three cooling rates: slow (1.38 ± 0.16 °C/h), medium (2.76 ± 0.32 °C/h), and rapid (5.52 ± 0.64 °C/h) cooling protocols to induce hypothermic dormancy at 14 ± 1 °C from 25 °C, followed by refrigerated transport. The result was that the muscle texture, color, and flavor of dormant shrimp were better than those of live shrimp, and the quality improved [68]. The key to gradient cooling-induced dormancy lies in balancing physiological homeostasis with product quality during the live transport of crustaceans, providing strong theoretical and practical support for the large-scale application of waterless live transport of *E. sinensis*.

In addition to temperature, salinity is a key factor regulating the osmotic balance and physiological homeostasis of *E. sinensis*. Drastic fluctuations in salinity force the organism to prioritize energy allocation for osmotic regulation, thereby restructuring its overall metabolic pattern [22]. Pre-treatment with acclimatization to moderate salinity prior to storage and transport can significantly improve the osmotic stability of crabs, effectively reduce the degree of ionic imbalance during exposure to anhydrous conditions, and simultaneously activate the organism’s endogenous antioxidant defense system, enhancing SOD activity and significantly alleviating oxidative stress-induced deterioration of meat quality. It is an effective pre-treatment method for improving the quality of live crabs during storage and transport [20]. Experiments on combined salt and alkalinity stress further confirmed a dose–response relationship for salinity stress: moderate salinity stress can maintain the organism’s osmotic homeostasis, whereas prolonged, high-intensity stress leads to persistent physiological disorders, significantly downregulating the activity of key antioxidant enzymes such as CAT, SOD, and GPx, increasing levels of the lipid peroxidation product MDA, and impairing ATP synthesis, ultimately inducing systemic metabolic dysfunction [93]. In addition, the rate of salinity decrease has a significantly different impact on physiological damage to the crabs. Compared with rapid salinity reduction treatments at 15‰/24 h and 30‰/24 h, slow gradient salinity reduction at 5‰/24 h and 10‰/24 h effectively maintained the organisms’ T-AOC and CAT activity, significantly reduced oxidative damage, and inhibited apoptosis, confirming that the rate of salinity change is a major factor amplifying stress-induced damage during storage and transportation [65]. In summary, the cascade regulatory pathway of “salinity signal transduction–osmotic pressure regulation–energy redistribution–antioxidant balance” precisely regulates the antioxidant levels and muscle quality of *E. sinensis*, forming a dual regulatory system that addresses both osmotic and oxidative stress and provides important theoretical support for the precise dynamic regulation of salinity during the post-harvest period.

### 5.2. Comprehensive Advantages: From Laboratory to Industry

Compared to traditional transport methods, the anhydrous live transport of *E. sinensis* demonstrates significantly enhanced multidimensional core application value through the synergistic application of gradient cooling technology. Under this transport model, crab survival rates reach 85% after 48 h, markedly exceeding the 65% achieved by traditional water-based transport, while the crab meat’s TVB-N content remains stable at 12.3 mg/100 g, below the industry freshness threshold standard. The retention rate of umami amino acids exceeds 90%, achieving precise preservation of product quality throughout the entire transport process [94,95]. Regarding resource conservation and cost management, waterless live transport reduces water consumption by 90% and energy expenditure by 30% [96]. In contrast, the low-temperature freezing and refrigerated processing and preservation system showed that under freezing storage conditions of −20 to −40 °C, the TVB-N of crab meat remained stable at 12.3 mg/100 g. In contrast, after 8 days of high-pressure processing and storage at 4 °C, the TVB-N in crab meat reached as high as 244.50 mg/100 g. During storage, the fatty flavor continued to diminish while the fishy odor gradually intensified. The effective storage period for this process does not exceed 6 days, and its freshness-preservation performance is far inferior to that of gradient-controlled, waterless live transport [91,97]. It is crucial to note that during the anhydrous live transport of *E. sinensis*, multiple environmental factors—including temperature and humidity fluctuations, mechanical vibration, DO depletion, elevated CO_2_ concentrations, and ammonia nitrogen accumulation—can induce compound stress effects that synergistically impair muscle quality [81]. Although this technology exhibits comprehensive advantages across core dimensions, including transport survival rate, meat quality preservation, cost-effectiveness, and environmental adaptability, the practical industrialization effects of these technical measures remain constrained by the cumulative impacts of environmental variables such as temperature and humidity fluctuations. This, in turn, affects the ultimate conversion of industrial economic benefits.

Compared to live transport of other crab species, live air transport of *Paralithodes camtschaticus* for 20 h elicited typical physiological responses such as hypoxia stress in the crustaceans [98]. Live transport of *Cancer pagurus* within polystyrene-insulated boxes filled with seawater-soaked straw, maintained under high humidity conditions, significantly extended survival time, slowed decay rates, and reduced transport costs [99]. Low-temperature transport of *Chionoecetes opilio* effectively reduces microbial spoilage rates [100]; for brown crabs, employing oxygenated water treatment at temperatures below 5 °C significantly improves survival rates during transit [101]. In summary, existing research on live crustacean transportation indicates that waterless transport effectively inhibits microbial spoilage rates, enhances transport survival rates, and reduces costs. Furthermore, integrating gradient cooling technology with this approach can further minimize physiological stress damage to the crustaceans. This provides new insights for optimizing live transport protocols for *E. sinensis* within simulated commercial environments.

### 5.3. Key Influencing Factors and Regulatory Strategies in Practical Applications

For freshwater live *E. sinensis* transported without water, it is necessary to induce them to enter a dormant state to reduce metabolism and thus prolong the survival time. Temperature gradient regulation is a key factor in this technology. This technical system employs multi-stage sequential temperature gradients to progressively lower environmental temperatures, driving a stepwise reduction in metabolic rate. This concurrently diminishes energy substrate consumption and stress response intensity, thereby enabling gradual adaptation to low-temperature conditions. This model can relieve acute stress caused by rapid temperature drop and reduce oxygen consumption and energy demand [102]. By reducing the basal metabolic rate, the survival time of *E. sinensis* will be prolonged [34]. Optimizing the cooling parameters can relieve oxidative stress and tissue damage during transportation and maintain the physiological stability of the crab. This, in turn, improves the transportation survival rate and product quality.

Industrialized breeding and live transportation technology for *E. sinensis* needs to carry out precise control for different scenarios. Take transportation time and the specifications of *E. sinensis* as core adjustment parameters. In the short-distance scenario where the transportation time is less than 24 h, adopt the compound control scheme of gradual cooling and moisturizing shavings (humidity 85%) to maintain a stable hibernation state. Meanwhile, the mechanical vibration intensity should be controlled to be less than 0.5 g to reduce the risk of physical damage [66]. Long-distance transportation (24–72 h) requires improved precision: phase-change materials stabilize the ambient temperature at 5 ± 1 °C. When juvenile crabs are less than 100 g, their metabolism is relatively high, and they need to be cooled at a gradient of 0.5 °C per hour and also need to be equipped with 90% humidity to maintain water balance. When adult crabs are more than 200 g, their stress tolerance is relatively strong, and they can withstand a cooling rate of 1 °C per hour and an optimal humidity of 80% [23,24]. The anhydrous live transportation technology deeply integrates gradient cooling, physiologically reduces oxidative stress and mechanical damage, and maintains the stability of the organism and muscle quality. The increasing demand for live aquatic products from consumers means that this technology system meets the needs of the long-distance transportation industry, enhances the market competitiveness of products, reduces transportation costs and water consumption, and has obvious ecological and economic synergetic effects.

### 5.4. Existing Challenges and Breakthrough Directions

Although the anhydrous live transport technology for *E. sinensis* has significantly improved survival rates during transit, multiple core challenges persist in its industrial-scale implementation. These manifest as stringent requirements for precision in gradient cooling and temperature control; substantial investment in equipment, technical resources and development; variations in the population’s environmental adaptability; and risks of disturbance during transport. These practical bottlenecks delineate key breakthrough directions for subsequent research.

The problem of insufficient temperature control accuracy is a relatively large issue. *E. sinensis* experiences obvious physiological stress from temperature fluctuations, such as a sudden, sharp rise in temperature during summer transportation. Such extreme temperature changes will awaken dormant crabs in advance, causing metabolic rebound and suffocation due to oxygen consumption. To address this, intelligent temperature control equipment maintaining deviations ≤ ±0.5 °C must establish stable microenvironments. In terms of hardware, it is necessary to reduce the physiological stress caused by temperature [90]. The mechanism of delayed rewarming and immune regulation requires further investigation as soon as possible. Studies have also shown that under short-distance transport without water, rehydration within 3 h alters the activities of key metabolic and immune enzymes (such as alkaline phosphatase, AKP; acid phosphatase, ACP; and aspartate aminotransferase, AST). Concurrently, the immune system undergoes self-regulation to form an immune barrier against external pathogens. This endogenous protective mechanism mitigates the damage caused by water-deprivation exposure to both the oxidative stress system and immune function. Gradual rewarming in water at 15 °C can increase the survival rate of crabs by 10%. The lack of standardized operation in verifiable technologies affects the application efficiency [103]. The lack of a standard transportation system and insufficient population adaptability are the main obstacles in the industry. There are obvious differences in the stress resistance of *E. sinensis* populations in different river basins. Current technologies, such as morphological characteristics, microsatellite markers, fatty acid composition, flavor components, and multi-element analysis, can achieve precise origin tracing [104]. However, a mature research framework is still lacking for developing tailored models that incorporate origin, size, grade, and transportation parameters. This deficiency hinders the application of technologies designed to balance universal applicability with case-specific precision.

At present, there are few studies on the vitality monitoring of *E. sinensis* in anhydrous transportation. However, there are relevant achievements in related technical fields: Before transportation, the non-contact and non-destructive Faster MSSDLite image analysis technology is used to collect the vitality data of crabs to ensure sufficient vitality. This technology enables large-scale, real-time, precise, and non-destructive analysis, and its overall performance in low-resolution images is superior to that of traditional technologies. However, the model lacks adaptability to complex scenarios, and image recognition accuracy is susceptible to interference from water vapor in low-temperature storage and transportation environments. It is difficult to directly apply this technology to long-distance storage and transportation without water, and further optimization is needed to enhance its practical performance in the field [105]. Regarding live transport monitoring, existing research has established a comprehensive monitoring platform for the entire live crab transport process. This platform integrates the hazard analysis and critical control point (HACCP) quality control system with the supply chain and information fusion technology. It can collect key environmental data, including temperature, relative humidity, oxygen, and ethanol levels, from inside storage and transport containers in real time. Combined with a cloud-based early-warning module that dynamically identifies stress risks, the system enables the visualization of energy metabolism during the transport of live crabs. The system’s detection accuracy correlates with temperature changes: the detection accuracy at 4 °C exceeds 99%; at 25 °C, the accuracy rate is 86% [90]. Drawing on the mature, intelligent storage and transportation system for aquatic products, the low-temperature storage and transportation system for live fish without water has been successfully commercialized. Under temperature-controlled conditions ranging from 0.5 to 2.5 °C, the survival rate of aquatic products consistently exceeds 96%. In addition, IoT terminals can collect environmental data in real time and automatically adjust transportation parameters such as temperature, humidity, and ventilation, thereby continuously mitigating oxidative stress damage caused by low temperatures and vibrations. This integrated smart system can further increase the survival rate of aquatic products at the point of delivery to 99.5%. However, the system is still in its early stages of development, and there is room to further strengthen precise monitoring and dynamic control across the entire supply chain to drive the optimization and upgrading of the smart logistics system for fresh aquatic products [29]. A vitality assessment could also draw upon dynamic monitoring and quality assessment methods in the fresh-product industry. By combining on-site analytical methods such as rapid spectral analysis and electronic noses to simultaneously assess the extent of protein oxidation and lipid deterioration, we can comprehensively improve survival rates and meat quality during transportation [106]. The introduction of digital tools such as AI-powered image recognition, multi-source sensor data fusion, and satellite remote sensing for environmental forecasting has further expanded the research directions and application boundaries of intelligent monitoring and quality assessment of fresh aquatic products [28]. Extracting technical insights and transportation models from the vitality monitoring system and applying them to other waterless transportation scenarios of aquaculture products may fill the technical gap in intelligent vitality monitoring in the field of *E. sinensis*. Cases related to *E. sinensis* are helpful for the development of an efficient and green anhydrous fresh-keeping technology system.

In summary, anhydrous live transport technology boasts broad application prospects in the logistics of *E. sinensis* through core equipment innovation, optimized regulatory processes, and tailored model development. Current core challenges (temperature control precision, cost management, and standardization) are key priorities for future research. These efforts will drive technological innovation and process upgrades, facilitating the transformation of this technology from laboratory research to a large-scale industrial application, which serves as an essential requirement for the industry.

## 6. Conclusions and Future Prospects

This review systematically elucidates the regulatory mechanisms by which holding tank technology and live transport systems maintain survival rates and commercial quality, centered on the *E. sinensis* “survival–quality–value” regulatory chain. Existing research has confirmed that key environmental factors, such as dissolved oxygen, water temperature, salinity, and ammonia nitrogen concentration, play a central regulatory role. These factors directly regulate oxidative stress, energy metabolism, and osmotic balance in crabs; through cascading effects, they alter their nutritional value and flavor characteristics, ultimately determining the economic value within the product supply chain. However, current research still has significant limitations: storage and transportation technologies are often based on studies of survival rates under constant optimal parameters, making it difficult for them to adapt to the complex realities of long-distance logistics and dynamic environmental fluctuations. Particularly during long-distance storage and transportation, sudden stressors such as abrupt temperature changes, transient hypoxia, and water quality deterioration frequently occur, leading to physiological and metabolic disorders, weakened immunity, meat quality deterioration, and the accumulation of harmful substances in the crabs, thereby posing food safety risks. At the same time, dynamic risk early-warning technologies covering the entire process are still in the preliminary exploration stage, while IoT sensing and non-destructive rapid detection methods remain largely at the conceptual design level, lacking practical simulation and validation under long-distance transportation conditions. Issues such as metabolic disorders, meat quality deterioration, and the accumulation of endogenous harmful substances in crabs under dynamic conditions cannot be effectively mitigated by existing single-measure control methods. There is a significant disconnect between theoretical models and actual storage and transportation practices, and insufficient capabilities for intelligent monitoring, precise control, and emergency intervention are hindering industrial upgrading.

Given *E. sinensis*’s poor adaptability to live storage and transportation, the difficulty in managing stress, potential food safety risks, and challenges in scaling up the technology for industrial application, future research should focus on real-world industrial scenarios and develop a comprehensive technical system centered on food safety that integrates monitoring, early warning, and intelligent control. This should focus on two key directions: Firstly, developing a multidimensional monitoring system that integrates real-time sensing with linked food safety indicators. Building upon precise tracking of environmental parameters such as temperature fluctuations, ammonia nitrogen dynamics, and DO levels, this system will concurrently monitor core food safety metrics, including oxidative damage markers in crab tissue, microbial content, and TVB-N. This will enable dynamic early warning of stress-induced food safety risks. Secondly, by integrating real-time IoT monitoring, end-to-end blockchain traceability, smart packaging sensors, and rapid non-destructive testing technologies, we have established an integrated smart management platform that links “physiological responses, environmental parameters, and quality changes.” This platform facilitates the further calibration of thresholds for live-preservation techniques—such as gradient cooling, salinity acclimatization, and green anesthesia—and establishes standardized storage and transportation protocols that balance stress resistance and food safety. Such an approach will provide comprehensive, precision-controlled food safety management throughout live transport, enhancing end-to-end food safety assurance capabilities for aquatic products from holding facilities to final consumption.

## Figures and Tables

**Table 1:** Summary of suitable range for key environmental parameters in the live storage and transportation of *E. sinensis*.

Environmental Parameters	*E. Sinensis* Sizes	Storage and Transportation Conditions	Result	Reference
Fast-cooling (FC): 7 °C CA treatment; Slow-cooling (SC): From 25 °C to 7 °C (3 °C/h)	100 ± 5 g	A plastic storage box (490 mm × 345 mm × 285 mm), with constant-temperature water	CA treatment boosts survival and inhibits muscle degeneration, with SC showing notable efficacy	[37]
Temperature (7.0–15.8 °C); relative humidity (44.0–58.8%)	150–160 g (Male *E. sinensis*)	Foam boxes (30 cm × 20 cm × 14 cm), replace the ice every 24 h	3 days of waterless storage keeps vitality, metabolism and muscle structure optimal	[39]
Water temperature (15 ± 0.5 °C); salinity (15%)	69.14 ± 2.24 g	A plastic aquarium (60 cm × 45 cm × 45 cm), fully aerated tap water and sea crystal	Higher salt concentration elevates serum osmotic pressure and reduces Na^+^/K^+^-ATPase activity	[22]
Water temperature (23.0 ± 1.0 °C); pH value (approximately 7.3); DO concentration (≥7.0 mg/L)	200 ± 15 g	Compound eugenol–triene anesthetic, anesthetic concentration 158.28 mg/L	Combined anesthesia boosts transport survival by regulating key metabolic pathways	[62]

**Table 2:** Summary of optimal ranges for various analytical parameters of e. sinensis under multifactorial stress.

Key Specifications	Optimal Values or Optimal Ranges for Key Parameters	Collaboration Parameters and Ranges	Result	Reference
Temperature	7 °C	Cooling Rate: 3 °C/h	Effectively protects muscle fiber structure, improves meat moisture retention and texture, and enhances vitality and survival rates	[37]
DO	9.5–11 mg/L	Water temperature: 17.92 ± 0.43 °C Salinity: 18.15 ± 0.34% pH: 7.29 ± 0.06	Promotes aerobic metabolism in the body, regulates lipid metabolism, and supplies energy for the crab’s vital functions	[41]
Ammonia Nitrogen	10.47 mg/L	Water temperature: 20.4 ± 1.4 °C pH: 7.69 ± 0.46	Low-ammonia exposure can upregulate phosphatase activity in the body and reshape immune, detoxification, and metabolic pathways	[18]
pH	6.5–9.0	Water temperature: 24 ± 1.0 °C DO: >7.5 mg L^−1^ Ammonia Nitrogen: <0.05 mg L^−1^	Effectively enhances the crabs’ antioxidant capacity, immune function, and tolerance to environmental stress	[19]
Salinity	18‰	Water temperature: 21 ± 1 °C pH: 7.6 ± 0.3	Significant improvements in the body’s osmotic regulation and resistance to oxidative stress	[58]

**Table 3:** Common temperature control techniques in live animal transport.

Temperature Control Processing Technology	Advantages	Disadvantages	Reference
Pre-cooling treatment technology	Enhance vitality recovery, relieve metabolic stress and inhibit metabolic physiological changes	Reduced pH acidifies hemolymph, accumulates ammonia nitrogen metabolites and aggravates oxidative stress damage in organisms.	[69]
Dynamic temperature control processing technology	Low temperature induces bodily dormancy, reduces metabolic activity and enhances antioxidant self-protection	Sharp temperature fluctuations trigger strong oxidative stress, lowering survival rates and impairing physiological quality indices	[70,71]
Cold anesthesia technology	Boosts vitality and survival rate markedly, alleviates oxidative damage and pH drop effectively, and preserves muscle fiber structure, water-holding capacity and texture properties	Crustaceans have a critical dormancy temperature range; persistent extreme deviation causes mortality	[37,72]
Gradient heating stress simulation technology	Moderate warming can enhance immune activity and improve survival rates	Temperature induces heat shock protein (HSP) production; prolonged heat impairs quality and cellular viability.	[73,74]

**Table 4:** Comparison of common live transport methods.

Live Transportation Methods	Advantages	Disadvantages	Practical Applicability	Reference
Anesthesia to preserve life	Reduced body metabolism, oxygen consumption, and ammonia excretion for safe and efficient live transport	Over-injections can cause safety issues and drug residues	Scientific laboratory procedures, long-distance transportation of goods, aquaculture, fishing and sorting, and invasive aquatic surgical procedures	[87,88]
Low temperature to preserve life	Reduced metabolism, oxygen consumption and ammonia excretion enable safe, efficient live transport	Oxidative stress caused by sudden drops in temperature makes long-term low-temperature storage and transportation impossible, resulting in significant limitations on storage and transportation duration	High-volume long-distance road transport, temporary storage of bulk shipments at seafood retail markets, and time-sensitive air transport of fresh produce	[23,89]
Provide oxygen to preserve life	Providing sufficient oxygen during transport significantly reduces economic losses in aquaculture production	Under multiple environmental stresses, such as water eutrophication and high temperatures, low dissolved oxygen levels tend to recur frequently, requiring the continuous operation and maintenance of aeration equipment	Regulating Water Quality to Maintain Fish Survival in High-Density, Large-Scale Aquaculture Ponds During Hot Seasons	[44]
Water purification to preserve life	Reduces CO_2_ and ammonia excretion, filters impurities and purifies water	Purification and packaging parameters vary depending on the type of crustacean, and continuous flow filtration systems increase packaging and logistics costs	Regulating Water Quality to Maintain Fish Survival in High-Density, Large-Scale Aquaculture Ponds During Hot Seasons	[21]

## Data Availability

Data will be made available on request.
